# Well-Paid Matrons

**Published:** 1919-07-19

**Authors:** 


### THE MATRONS' AND SISTERS' DEPARTMENT.

### WELL-PAID MATRONS.

The position of matron in an institution holds within itself a strong power of attraction. The satisfaction which it imparts is altogether apart from its financial value. It is a position of great influence. It demands self-devotion while supplying the worthiest possible objects for the exercise of this high quality. It is of the order of work which women in all ages have delightedly accepted. Its hourly opportunities for service continually exceed the written obligation. And the matron who, while accepting her full responsibilities as the paid officer of the institution, does not dedicate herself in the truly voluntary spirit of vocation to the business in hand has not attained to the heights of her profession. In principle matrons are, in fact, voluntary workers, and the payment accorded to them was for many a long day rather in the nature of an honorarium than a fair wage for hard work accomplished. That this system is not extinct may be seen from the report of the Salaries Committee, where it is recorded that matrons' salaries in large institutions sink in certain cases as low as £45, £55, £60, or £80.

We owe to this selfless spirit of service some of the historic founders of the nursing profession. Yet now their work is accomplished we can recognise that the system which accepted their services and left them in old age to something like penury was cruel. Whether good or bad, it is dying fast. At present the interests of the nursing service are closely bound together and in the evolution of its members from candidate to matron, the ill-payment of any section in the ascending scale reacts for harm on all. Yet the halo of the old tradition that the matron dedicates herself to work which holds strong attractions for her over and above that of salary, still appears to hang around many of these posts. Can it be that employers have insensibly come to believe that they are actually conferring a favour in electing their matron by opening up to the woman of their choice an opportunity for service which outbids in her eyes any material advantages in the post?

There is a very curious point in the investigations on salaries published by the Salaries Committee of the College of Nursing. This Committee had the opportunity of contrasting the salaries paid in thirty-four Provincial Poor-Law Infirmaries. The average matron's salary paid in eleven of these institutions containing from 200 to 300 beds was £65½; the the average salary paid in thirteen institutions containing 100 to 200 beds was £75½; and as the number of beds decreased the rate was again raised so that in two institutions containing less than 100 beds the salary was £80. It is perfectly clear that the value of the experience gained in the larger institutions and the uncovenanted attractions of these posts were reckoned on by the Guardians as a reasonable excuse for getting the matron cheap. In other words, the matrons in these institutions are doing voluntary work like the Guardians themselves. They receive an honorarium it is true, but not the value of their services.

This part-payment of matrons is a very bad thing for all the other women employed in the institution. The writer well remembers an occasion when the engagement of a chef was under discussion in a London institution suffering under the wastefulness of bad cooking. The proposal was brushed aside because a properly qualified cook would require a higher salary than the matron then received. Notoriously this consideration has debarred some institutions from engaging trained and certificated housekeepers. Thus starting with impossible theories as to the payment of the matron, managers have been driven to writtle down all the salaries to make their scheme appear decent.

Everyone knows that matrons of the right sort are not to be bought for money. And in some minds a doubt arises whether higher pay will have any effect in increasing the number of women able and willing to dedicate themselves to the multiple responsibilities of their posts. In making the matronship a business proposition, are the right women going to be elbowed out? We believe the misgiving to be groundless. The immediate effect of increasing matrons' salaries has been and will be to secure for this branch of the service a very large proportion of women who cannot afford to work all their lives for a pittance. It has long been an axiom in business circles that in actual money value high salaries to competent directors are the truest economy. How many admirable potential matrons drift away from institutional life to private nursing or spend their small capital on nursing homes for financial reasons. The cheese-paring policy through which managers and guardians have hitherto exploited the devotion of their matrons is at last disappearing. When it has vanished the devotion will still be left.

